# The Modulatory Effect of Cerebrovascular Burden in Response to Cognitive Stimulation in Healthy Ageing and Mild Cognitive Impairment

**DOI:** 10.1155/2019/2305318

**Published:** 2019-08-06

**Authors:** Charlotte Bentham, Matteo De Marco, Annalena Venneri

**Affiliations:** ^1^Neuropsychology Department, Department of Psychological Services, Sheffield Teaching Hospitals, Sheffield, UK; ^2^Department of Psychology, University of Sheffield, Sheffield, UK; ^3^Department of Neuroscience, University of Sheffield, Sheffield, UK

## Abstract

**Background:**

Cerebrovascular burden is a common pathology in mild cognitive impairment (MCI) and Alzheimer's disease (AD), with an additive impact on cognitive functioning. Despite being proposed as a potential moderator of cholinesterase inhibiting drug therapy, there is a paucity of evidence investigating the impact of cerebrovascular pathology on responsiveness to cognitive interventions.

**Method:**

The current study uses neuropsychological, neurostructural, and functional connectivity indices to characterise response to a cognitive stimulation paradigm in 25 healthy ageing and 22 MCI participants, to examine the hypothesised detrimental effects of concurrent vascular pathology.

**Results:**

In both healthy ageing and MCI, increased levels of vascular pathology limited the potential for a neuroplastic response to cognitive stimulation. In healthy ageing, participants with lower levels of vascular burden had greater functional connectivity response in the target posterior default mode network. Those with low levels of vascular pathology in the MCI cohort had increased functional connectivity of the right insula and claustrum within the salience network. Burden did not, however, predict cognitive or neuroanatomical changes.

**Conclusions:**

The current research evidences the modulatory effect of cerebrovascular pathology in interventions aimed at re-establishing network connectivity to prevent cognitive deterioration and delay the transition to the dementia stage of AD. Examination of co-occurring vascular pathology may improve precision in targeting treatment to MCI candidates who may respond optimally to such cognitive interventions.

## 1. Introduction

The prevention and treatment of Alzheimer's disease (AD) is a primary target of research as AD is set to rise exponentially with increased population growth and life expectancy [1].

In the absence of an effective form of treatment for the prodromal or even preclinical stages of this disease, cognitive interventions remain a viable option. However, due to variability in the content and theoretical underpinnings of cognitive interventions, they have received inconsistent support [2, 3]. De Marco et al. [4] created a programme of computerised cognitive stimulation specifically designed to upregulate functional connectivity within the brain default mode network (DMN), the network active when individuals are engaged in internally focused tasks and in which abnormalities of activity have been found in AD [5, 6]. De Marco et al.'s programme was validated in a cohort of healthy seniors. In those with MCI, this cognitive training regime was associated with increased connectivity within the left parietal regions of the posterior DMN, and upregulation in this region in turn corresponded with a moderate, yet positive, change in cognitive performance [7].

With the high degree of heterogeneity in the type and degree of symptomatic response to both pharmacological and cognitive therapeutic approaches, identifying moderating factors influencing response will allow increased precision in targeting treatments to those individuals who will derive the greatest benefit. One potential moderator is cerebrovascular burden, which can be seen as bilateral, patchy, or diffuse areas of hyperintensity on T2-weighted magnetic resonance imaging (MRI) scans involving the periventricular and *centrum semiovale* white matter [8]. Fluid attenuation inversion recovery (FLAIR) and proton-density MRI acquisitions are also commonly included in radiological protocols for a similar purpose. Compromise of white matter integrity leads to a less effective electrical impulse conduction along the white matter fibre tracts and, therefore, interferes with the normal functioning of the brain [9].

Cerebrovascular burden prevalence in MCI brains ranges from 70% to 100%, and the distribution of lesions is more extensive than in the healthy ageing population [10]. The presence of vascular pathology contributes to accelerating the progression from MCI to AD [11]. Evidence suggests that severe white matter lesions are associated with impaired cognitive abilities, specifically within the domains of executive functioning, attention, processing speed and global cognition [12, 13]. fMRI research demonstrates a negative association between increased white matter damage load and resting-state abnormalities in the DMN [14].

Pantoni et al. [15] confirmed that a computerised cognitive training programme can result in improved selective cognitive performance and functional connectivity compared to controls in MCI participants with comorbid small vessel disease. However, only one recent study has investigated the differential cognitive response of computerised cognitive training paradigms in those with MCI according to accumulation of white matter pathology. In an exploratory analysis, Djabelkhir-Jemmi et al. [16] found that a three-month programme of cognitive exercises resulted in greater improvements in category fluency and paired associate learning in those with lower levels of white matter pathology.

The current paper is aimed at adding to the literature investigating the relationship between vascular burden and treatment response to cognitive training paradigms, whilst also attempting to overcome some of the limitations of the methodology used in the field. Firstly, the semiquantitative visual rating scales [17, 18] predominantly used in this field to measure cerebrovascular load are limited in that they are less sensitive to small group differences [19] and may have ceiling effects leading to under-representation of the severity of white matter lesions [9]. Volumetric quantification methods adopted in the current study have been suggested to offer an unbiased measurement of lesion load [20]. In the reported study, a comprehensive neuropsychological battery was used to measure cognitive change as a function of treatment, which is less likely to mask treatment effects due to limited psychometric properties than commonly used cognitive screening tools. In addition, treatment response was characterised using imaging variables: fMRI to assess neuroplastic increases in functional connectivity and structural MRI to explore any associated morphological modifications. Imaging variables are able to detect response to both cognitive and pharmacological interventions which, due to the short duration of treatment, may not be sufficient to result in observable clinical changes in cognitive assessment [21].

## 2. Method

### 2.1. Participants

The current research was based on secondary analyses of data collected in previous studies [4, 7]. A total of 47 participants were included in the analysis, comprising of 22 participants with MCI and 25 healthy elderly participants (see Table 1 for details).

### 2.2. Network-Based Cognitive Stimulation

All participants in the sample were enrolled in a programme of computerised cognitive stimulation, specifically designed to upregulate the DMN. A comprehensive description of the package is outlined in De Marco et al. [4]. The tasks made demands on a range of cognitive domains including semantic retrieval, logical reasoning, response time, and proper name retrieval, with the aim to induce coactivation of the subcomponents of the DMN regions. The participants were required to complete 20 sessions of the package within 20-25 days, with each session lasting between 60 and 90 minutes.

### 2.3. Cognitive Testing

A neuropsychological assessment was conducted at participant enrolment to the study in order to improve characterisation of the sample, as well as establish baseline cognitive functioning. The battery included a range of tasks tapping diverse cognitive domains, including measures of global cognition, verbal (category and letter) fluency, short-term and working memory, long-term memory, executive functioning, abstract reasoning, visuoconstructive abilities, and attention (see Table 2). The neuropsychological assessment was also repeated on completion of the cognitive stimulation package.

Cognitive performance of the healthy ageing sample was used as normative data to characterise the cognitive performance of the MCI cohort. Using Petersen's [35] criteria, scores falling 1.5 standard deviations below (or above, in the case of the Stroop test) the mean healthy ageing score in a task were considered impaired. Based on this classification, the MCI participants were distributed as follows: one amnestic single domain, five nonamnestic single domain, 14 amnestic multidomain, and two nonamnestic multidomain [35].

### 2.4. MRI Acquisition

An MRI protocol including both structural and functional sequences was acquired on a 1.5 T Philips Achieva System, at both baseline and following the completion of the cognitive stimulation paradigm. The following brain MRI sequences were included in the scanning protocol: T1-weighted sequence type: Turbo Field Echo 3D, repetition time: 7.4 ms, echo delay time: 3.4 ms, flip angle: 8°, voxel dimension: 1.1 × 1.1 × 0.6 mm^3^, gap: 0.6 mm, field of view: 250 mm and matrix size: 256 × 256 × 124. Resting-state echo planar sequences were acquired in two runs of 120 volumes each. Acquisition details were as follows: number of slices: 20; volume acquisition details: axial orientation, bottom-up contiguous, and gapless; repetition time: 2 s; echo delay time: 50 ms; flip angle: 90°; voxel dimensions: 3.28 × 3.28 × 6.00 mm^3^; and field of view: 230 mm. A two-dimensional T2-weighted FLAIR magnetic resonance imaging sequence was also acquired (voxel size: 0.75 mm × 0.75 mm × 4.94 mm; TR: 8 s; matrix size: 320 × 320 × 20; and scan duration: 192 s). Images were checked by a neuroradiologist to rule out the presence of any abnormality meeting the exclusion criteria.

### 2.5. MRI Pre-processing Procedures

The pre-processing and modelling of neuroimaging data were completed using Statistical Parametric Mapping 12 (SPM-12, Wellcome Centre for Human Neuroimaging, London, UK) and additional toolboxes, using MATLAB (R2014a).

#### 2.5.1. T1-Weighted MRI Images

Voxel-based morphometry (VBM) pre-processing was carried out on anatomical scans. This included manual reorientation and coregistration of the scans taken before and after treatment to ensure uniformity. Next, probabilistic segmentation of white and grey matter maps from other tissue classes was conducted. Native-space volumes of grey matter, white matter, and cerebrospinal fluid were extracted using the “get_totals” script to calculate total intracranial volume and tissue ratios. The segmented maps were then normalised to a European brain template and finally smoothed with an 8 mm full width at half maximum Gaussian kernel. The pre-processed grey matter maps were used in statistical modelling.

#### 2.5.2. FLAIR Images

The Lesion Segmentation Tool in SPM-12 was used to estimate the volume of white matter hyperintensities (WMH) to give an index of vascular burden. Initially developed to segment white matter lesions in multiple sclerosis [36], it has also been utilised in research with healthy ageing, MCI, and AD participants [20, 37, 38]. This automated tool determines the three tissue classes from a T1-weighted MRI image that is coregistered with the FLAIR image. The FLAIR intensity distribution of each tissue class is computed to detect outliers, which are then probabilistically assigned to white matter, grey matter, or lesion classes [36]. A native space lesion map is created. A threshold of 0.3 was used. Native space lesion volume for each participant was then extracted.

The quantification of native space lesion volume was then divided by the total intracranial volume and then multiplied by 100 to express white matter hyperintensity volume as a percentage of intracranial volume. This served to obtain a white matter lesion (WML) index.

#### 2.5.3. Functional MRI

fMRI scans were processed using a standardised procedure for each participant, for scans obtained both preceding and on completion of the stimulation intervention. A slice-timing correction was applied to align the acquisitions temporally. Spatial realignment of the volumes was then conducted and in-scanner motion examined. One participant from the MCI sample was excluded from the analysis of fMRI scans due to excessive motion that disrupted network signal. Following this step, all runs were normalised and registered to the SPM template. A band-pass filter (0.01 to 0.1 Hz) was used to discard any blood oxygen level-dependent (BOLD) signal noise, e.g., from cardiorespiratory rhythms and scanner drift. Finally, scans were smoothed with a 6 mm full width at half maximum Gaussian kernel.

**Figure 1:**
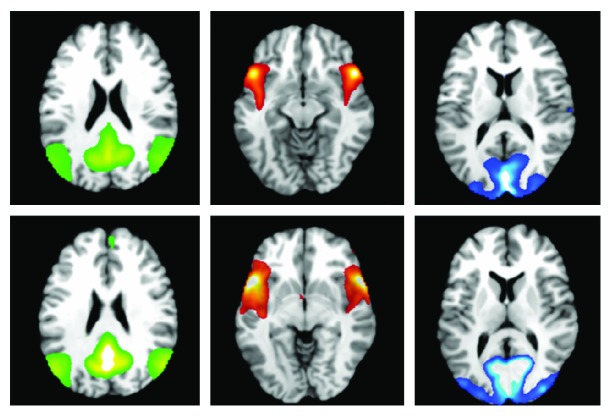
Mean fMRI network components extracted for analysis for healthy ageing and mild cognitive impairment participants. Top row: healthy ageing sample, bottom row: MCI sample. Networks extracted (from left to right): posterior DMN, salience network, and visual network.

To identify the neural network of interest, the posterior DMN, an independent component analysis (*ICA*) was used, computed using the GIFT toolbox (v1.3i, http://mialab.mrn.org/software/gift/). The *ICA* separates latent sources of variability (components) that are measured across the entire field of view. With this method, the map of each component is interpreted as a neural network. Due to differences between the MCI and healthy elderly participants, two separate *ICA*s were carried out, for each group individually. For the healthy elderly group, 25 (participants)-by-two (timepoints) runs were included. For the MCI group, 21 (participants)-by-two (timepoints) runs were included. For both *ICA*s, the number of components to estimate was set to 20. Two raters collaboratively identified the posterior DMN from the network components computed from the *ICA* for each group. In addition, two alternative networks were extracted: the salience network, as an anticorrelated network to assess the potential for compensatory network upregulation, and the visual network, as a non cognitive methodological control. By carrying out this procedure, components affected by non neural sources of variability such as in-scanner motion were discarded. The mean network components for the healthy ageing and MCI groups are illustrated in Figure 1.

A second set of *ICA*s was also conducted, extracting the three networks of interest at baseline from all participants (healthy ageing and MCI), in order to examine baseline differences in network expression between the two diagnostic groups. Similarly, these processing steps were also carried out for the post-treatment scans to run *post hoc* analyses.

### 2.6. Statistical Analysis

All statistical analyses were computed using SPSS Statistics (version 24) and SPM 12. Independent-sample *t-tests* (Tables 1 and 2) revealed significant differences in demographic, neurostructural, and neuropsychological variables between the healthy ageing and MCI groups. It was, therefore, decided that statistical analyses would be run separately for each group.

#### 2.6.1. Categorising Vascular Burden

To model appropriately treatment response and vascular burden simultaneously, a two-way full-factorial design was used. This required the classification of WML volume into a categorical variable: high and low WML groups. For the MCI and healthy ageing groups separately, the median WML volume was calculated and then used to divide each group into high and low WML subgroups for subsequent analysis.

#### 2.6.2. Neuropsychological Response

Cognitive tests were maintained as separate variables in order to establish if there were a differential effect on cognitive domains, as vascular burden has been evidenced to attenuate performance specifically on tasks of processing speed, immediate and delayed memory, executive functions, and indices of global cognitive functioning [12]. It was expected that due to ceiling effects observed in a number of the psychometric tests, a range of cognitive task scores would be non normally distributed. Therefore, following the testing of assumptions for parametric statistics, two procedures were utilised for analysis of neuropsychological response. For those variables meeting the assumptions of parametric testing, two-way mixed *ANOVA*s were used to test the interaction between WML group and treatment; main effects were also examined and reported. For those violating the assumptions required for the use of parametric *ANOVA*, an alternative method was established in which cognitive response was computed by calculating a difference score between the baseline and follow-up performance. *Mann-WhitneyUtests* were then used to explore any differences between the high and low vascular burden groups in cognitive response. To account for multiple comparisons, a Bonferroni-corrected significance level was used for these analyses.

#### 2.6.3. Neurostructural Response

A two-way full-factorial design was used to model the vascular burden-by-timepoint interaction on neurostructural response to treatment. The hypothesised interaction contrast term stipulates that those participants with lower levels of vascular burden will demonstrate more neurostructural response to treatment than those with high levels of vascular burden. The “inverse interaction” contrast, positing that the high vascular burden group would exhibit greater treatment response than the low group, was also tested as a methodological control. An uncorrected *p* value was set to *p* < 0.01 and peak coordinates were only reported as significant if they survived a *p* < 0.05 family-wise corrected *t*-statistic at a cluster level. Significant peak coordinates were converted into Talairach stereotaxic space (http://imaging.mrc-cbu. http://cam.ac.uk/downloads/MNI2tal/mni2tal-m) and interpreted using the Talairach Daemon Client [39, 40].

#### 2.6.4. Functional Connectivity Response

Mirroring the procedure used in the neurostructural analyses, a two-way full-factorial model was used, with vascular burden category and timepoint (treatment effect) as independent variables and the extracted fMRI networks as the dependent variables. The same interaction contrasts were maintained. A cluster extent of 300 contiguous voxels was selected, and a *p* value was set to *p* < 0.01, uncorrected. Again, peak coordinates were only reported as significant if they survived a *p* < 0.05 family-wise corrected *t*-statistic at a cluster level. The same procedure was used for interpreting the clusters using the Talairach Daemon Client.

## 3. Results

Table 3 shows that for each diagnostic group, age and years of education did not significantly vary between the high and low WML groups. Age and years of education did also remain constant across treatment, therefore, negating the necessity to control for these variables in subsequent analyses.

### 3.1. Neuropsychological Response

Assumption testing for the two-way mixed *ANOVA*s was carried out. Normality was largely supported by the *Shapiro-Wilk test* (*p* > 0.05); however, cognitive subtests with low variation in scores or with ceiling effects (Token task, Raven's progressive matrices, Rey complex figure copy, Stroop—error interference, Corsi test, digit span forwards and backwards, and naming) were shown to be non normally distributed, as anticipated, and therefore were analysed using a non parametric procedure.

Table 4 shows the results of the two-way mixed *ANOVA*s. There was no significant interaction between WML and treatment in any of the cognitive subtests used to characterise treatment response, even before Bonferroni correction of the significance level. Similarly, there was no significant main effect of WML on cognitive performance. In the healthy elderly sample, a significant effect of treatment was found in the phonemic fluency and prose immediate and delayed memory subtests, withstanding Bonferroni correction. No treatment effect, reaching the corrected threshold, was found in the MCI sample. The assumption of homogeneity of covariance was assessed using *Box's M*; violations of the assumption were reported for letter and category fluency in the healthy ageing cohort and Stroop time interference and prose immediate memory subtests in the MCI cohort. This forewarned of the caution needed during interpretation of findings. For the cognitive subtests that were non normally distributed, *Mann-WhitneyUtests* evidenced no significant difference in change in cognitive performance, between baseline and retest, between the low and high vascular burden groups (Table 5).

### 3.2. Neurostructural Response

The two-way full-factorial model showed no significant clusters withstanding the significance constraints stipulated, for either of the opposing directional contrasts. This suggests that vascular burden does not influence neurostructural response to cognitive stimulation treatment.

### 3.3. Functional Connectivity Response

#### 3.3.1. Baseline Network Comparison

Results of the independent-sample *t-tests* revealed a significant between-group difference in the DMN at baseline between healthy ageing and MCI participants. Specifically, the DMN component of healthy controls was significantly more expressed than that of patients in the left mediotemporal lobe including the hippocampus. Furthermore, a non significant trend was observed in the right mediotemporal lobe, precuneus, and medial prefrontal cortex. This pattern of differences is typically observed in a group of patients who are affected by AD. There were no baseline differences between the MCI and healthy participants in the connectivity of the salience network and the visual network. Overall, no trend suggesting differences in network-to-network interaction (e.g., DMN-visual network) emerged between patients and controls.

#### 3.3.2. Default Mode Network

**Figure 2:**
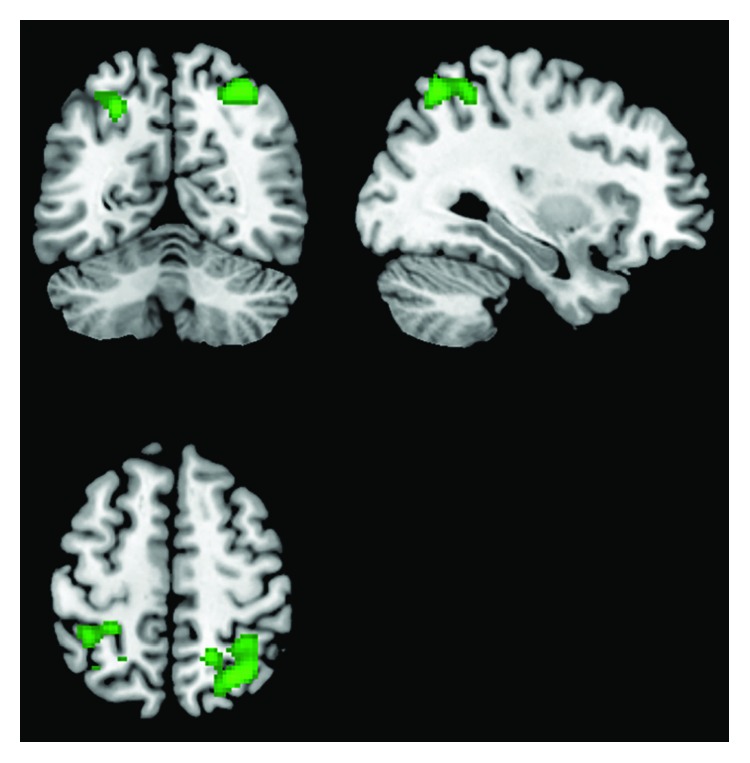
The effect of the interaction of WML and treatment on increases in the functional connectivity of areas of the posterior DMN in the healthy ageing sample.

The healthy controls exhibited a significant interaction between vascular burden and timepoint (treatment) in posterior DMN connectivity (see Table 6 and Figure 2). The increases in bilateral functional connectivity in those with low levels of WML were limited to the functional hubs of the posterior DMN, including the precuneus, as well as in the inferior parietal lobule component of the medial temporal substream of the DMN [41], including the postcentral gyrus. No significant clusters reached threshold for the MCI sample in the posterior DMN or for either of the inverse interaction contrast analyses.

#### 3.3.3. Salience and Visual Networks

**Figure 3:**
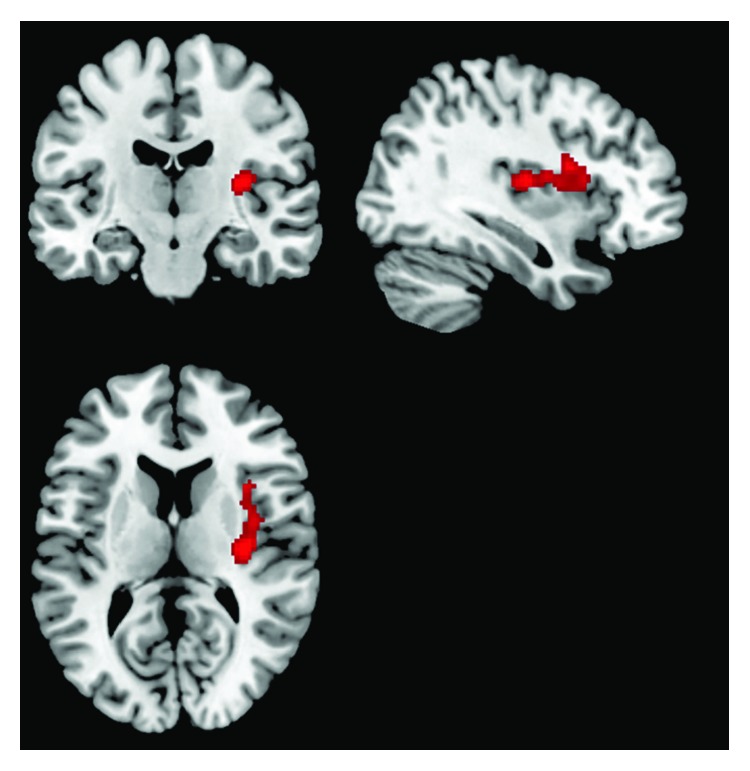
The effect of the interaction of WML and treatment on increases in the functional connectivity of areas of the salience network in the MCI sample.

A significant interaction between vascular burden and timepoint (treatment) was revealed for the MCI cohort within the salience network. MCI participants in the low vascular burden group demonstrated increased functional connectivity in the insula and claustrum components of the salience network compared with those in the high vascular burden group (see Table 6 and Figure 3). These findings were not replicated in the healthy ageing sample. No clusters maintained significance after thresholding for the analyses in the visual network or for any of the inverse interaction analyses conducted.

#### 3.3.4. *Post Hoc* Analyses

An exploratory *post hoc* analysis was conducted to study the interplay between WML and cognitive reserve (CR; education served as the cognitive reserve proxy in this study) on functional connectivity changes within the DMN and salience network in response to the cognitive stimulation intervention. Maintaining the two diagnostic groups (healthy adults: *n* = 25; MCI patients: *n* = 21) did not allow for division into four sufficiently sized sub-samples based on each combination of CR and WML levels. Therefore, the two diagnostic sub-groups were combined into a global sample (*n* = 46), and a global median split for both CR and WML was calculated, resulting in approximately equitable division of participants. The two networks were first recalculated with an *ICA* inclusive of the entire cohort. A 2 × 2 (time-by-WML) mixed-design factorial model for participants with low CR and, separately, for participants with high CR was then conducted. The results demonstrated that WML has an impact among participants with low CR only: those with low WML showed significantly more test-retest increases in DMN functional connectivity in a cluster located in the middle cingulate gyrus. No interaction was observed in the sub-group of participants with high CR.

## 4. Discussion

The current study demonstrates that in both healthy ageing and MCI, vascular burden is associated with a diminished functional connectivity response. Higher levels of compromise to white matter integrity attenuated functional connectivity increases in areas of posterior DMN in healthy ageing individuals and in the insula and claustrum areas of the salience network in those with MCI.

One potential mechanism by which vascular burden may moderate functional connectivity response to treatment is through decreased brain reserve and diminished potential for a neuroplastic response. Increases in the strength of resting-state functional connectivity are believed to be largely associated with increased strength of microstructural connections between the same regions [42]. As individuals with higher levels of vascular burden will have less viable neural reserve tissue available to facilitate this structural alteration, less functional connectivity alterations will be observed. Secondly, compromise to white matter microstructure may have a direct impact on connectivity of the posterior DMN. Supporting this suggestion, Teipel et al. [43] found that functional connectivity across the DMN is based on a distinct pattern of anatomical connectivity within the cerebral white matter (specifically, the cingulate bundle and fibre tracts connecting the posterior cingulate with lateral temporal lobes, medial temporal lobes, and precuneus). Therefore, white matter lesions within these white matter tracts may lead to attenuated connectivity within the network. Clarifying the mechanisms underlying the identified relationship would require examination of the white and grey matter microstructure using Diffusion Tensor Imaging, which is more sensitive to detecting cross-sectional alterations in white matter [44] and was not available as part of the imaging protocol in the current study.

Diminished treatment response in the DMN was not replicated in participants with a diagnosis of MCI. One potential explanation for this inconsistency posits that the DMN is pathologically down-regulated in individuals with MCI, representing the effects of ongoing early neurodegeneration [45]. Research suggests that areas of the DMN are pathologically down-regulated in MCI compared to healthy controls, especially in areas such as the precuneus [46, 47], right inferior parietal lobe [48], bilateral superior parietal lobes [45], left fusiform gyrus [48], and the precentral and inferior frontal gyrus of the right prefrontal cortex [45]. Therefore, in the MCI sample, the presence of early neurodegenerative pathology may take precedence above vascular burden in attenuating treatment response in the DMN, compared to healthy controls where neurodegeneration is not found.

The current study demonstrates that, in MCI, high levels of vascular burden limit a functional up-regulatory response within the salience network. This result extends the findings of De Marco et al. [38] who observed that, in healthy ageing participants, WML accumulation induces a neuroplastic up-regulation of the salience network within the right parietal cortex. In our MCI sample, those with low levels of vascular burden showed increased levels of recruitment of the right insula and claustrum. There are a number of potential reasons that these areas may have been up-regulated as a function of treatment. Firstly, evidence suggests that the right insula is functionally connected to frontal regions implicated in executive functioning and aids in the coordination and evaluation of task performance [49] and, therefore, may demonstrate increased frontal executive involvement when memory networks are depleted. Secondly, evidence supports that the DMN is anticorrelated with task-related networks [50]. One suggestion has been that Von Economo neurons, which are found predominantly on the right side of the brain and are abundant in the insula and claustrum, could facilitate switching between the DMN and task-related networks [50, 51]. In those without vascular damage, higher levels of functional connectivity in this area could, therefore, suggest increased switching between anticorrelated networks and the DMN, due to the pathological disconnection within the DMN in MCI.

No changes in neuroanatomical or cognitive indices of treatment response were observed in the current study. Using the same treatment paradigm, De Marco et al. [7] showed a trend, not reaching significance, in cognitive treatment response, positing that a longer intervention duration and larger sample sizes would be required to induce increases that would be measurable with neuropsychological assessment. Therefore, the limited variability in cognitive response may limit the power when assessing moderating variables. The functional connectivity increases demonstrated in the current study are posited to be associated with microstructural changes in the brain. Therefore, it would be logical to expect that vascular burden would also be predictive of neurostructural change. However, the assessment of grey matter changes using voxel-based morphometry, which is better deployed to detect macrostructural change, will be insufficient to characterise these small modifications.

Two important mitigating factors in the relationships between cerebrovascular pathology and treatment response are cognitive reserve and brain reserve. Brain reserve models postulate that individuals with greater measures of brain size, neural counts, or synaptic density can sustain higher levels of pathology before clinical manifestation becomes evident [52]. Inverse relationships have been reported between WML volume and brain volumes [20]. High cognitive reserve, on the other hand, facilitates better coping with brain pathology through some form of active compensatory strategy [53]. Dufouil et al. [54] found that cognitive reserve modulates the consequences of WML on cognitive performance. Our exploratory *post hoc* analysis suggests that the impact of WML is much more pronounced in participants with low levels of cognitive reserve, with those with low WML demonstrating significantly greater increases in DMN functional connectivity following cognitive stimulation intervention. Future research is needed to explore further how these variables may mitigate some of the detrimental effects of WML on treatment response.

It is important to note methodological limitations of the current study. Categorisation of the sample based on median volumes of WML reduces the variability and is not as sensitive to discrete group differences but is arguably less subjective than rating scales used predominantly throughout the literature. A further consideration is the use of a global measure of vascular burden, which does not maintain anatomical distinctions, which could theoretically have differential effects on response to treatment. The final consideration is the method used to characterise WML using lesion segmentation of white matter hyperintensities. Although similar in appearance on imaging, histologically, there is a distinction in aetiology between lesions [55]. Theoretically, algorithms deployed by the Lesion Segmentation Tool [36] should extend to characterisation of ischaemic lesions; however, it is unclear whether these algorithms will perform sufficiently well when the lesions are small, diffuse, or otherwise irregular in shape or intensity, which are characteristics of the subtle or emerging ischaemic lesions [56] that are the variable of interest in the current study. However, the advantage of this method is that it provides an unbiased measurement of lesion load and has been found to achieve good agreement with manual rating methods (*R*^2^ = 0.93, [20]).

## 5. Conclusion

The current study found that, in both healthy ageing and MCI, increased levels of vascular pathology limit the potential for neuroplastic response to cognitive stimulation. The current research evidences the modulatory effects of cerebrovascular pathology in interventions aimed at re-establishing network connectivity to prevent cognitive deterioration and delay the transition to the dementia stage of Alzheimer's disease. Examination of co-occurring vascular pathology may improve precision in targeting MCI candidates who may respond optimally to such cognitive interventions. Future research is required to replicate these findings and to clarify the mechanisms underlying the relationship between cerebrovascular pathology and treatment response using microstructural imaging techniques.

## Figures and Tables

**Table 1:** Demographic and neurostructural differences between the healthy ageing and MCI groups.

	Mean (SD)		*p* difference
	Healthy ageing	MCI	
*Demographic variables*			
Age	67.64 (7.35)	73.86 (5.21)	*p* = 0.002
Years of education	10.52 (4.12)	8.50 (3.65)	*p* = 0.084
Gender (m/f)	9/16	12/10	*p* = 0.202
*APOE ε*4 carriers (*N*, %)	5 (20%)	7 (33.3%)	*p* = 0.354
*Neurostructural volumes (ml)*			
Grey matter	586.12 (53.46)	532.88 (51.07)	*p* = 0.001^∗^
White matter	435.30 (42.87)	423.62 (55.46)	*p* = 0.421
CSF	351.71 (77.72)	451.34 (96.46)	*p* < 0.001^∗^
Total intracranial volume (TIV)	1373.12 (117.94)	1407.84 (157.45)	*p* = 0.393
WMH (ml)	3.30 (5.41)	8.58 (9.32)	*p* = 0.026
WMH/TIV (%)	0.24 (0.40)	0.60 (0.64)	*p* = 0.031

Means and standard deviations are indicated for all continuous variables. Proportional values are indicated for gender and number of *APOE ε*4 carriers. Group differences were calculated with independent-sample *t*-tests or *χ*^2^*tests* (gender and *APOE* genotype). Note: ^∗^ means significant when corrected for multiple comparisons (*p* < 0.0019).

**Table 2:** Baseline neuropsychological differences between the healthy ageing and MCI groups.

	Mean (SD)		*p* difference
	Healthy ageing	MCI	
Raven's progressive matrices [22, 23]	30.36 (4.66)	25.59 (6.71)	*p* = 0.006
Phonemic fluency [24]	32.76 (13.09)	28.27 (10.87)	*p* = 0.211
Semantic fluency [24]	43.16 (10.91)	28.55 (8.25)	*p* < 0.001^∗^
Digit cancellation [25]	54.04 (4.98)	45.00 (9.91)	*p* = 0.001^∗^
Similarities [26]	20.56 (4.36)	19.09 (4.62)	*p* = 0.268
Token task [25]	34.77 (1.78)	33.21 (2.54)	*p* = 0.019
Rey figure—copy [27–29]	32.28 (3.92)	27.96 (6.92)	*p* = 0.010
Rey figure—memory [27–29]	16.38 (6.10)	7.82 (4.88)	*p* < 0.001^∗^
Stroop—time interference [30, 31]	19.14 (7.01)	43.59 (29.60)	*p* = 0.001^∗^
Stroop—error interference [30, 31]	0.62 (1.20)	4.00 (6.66)	*p* = 0.028
Digit span—forwards [32]	6.12 (0.88)	5.45 (0.67)	*p* = 0.006
Digit span—backwards [32]	4.28 (1.10)	3.64 (0.73)	*p* = 0.021
Corsi span [32]	4.84 (0.90)	4.23 (0.61)	*p* = 0.010
Prose—immediate memory [33]	8.76 (2.89)	6.05 (3.14)	*p* = 0.003
Prose—delayed memory [33]	11.80 (4.35)	6.91 (4.29)	*p* < 0.001^∗^
Paired associate learning [33]	12.92 (3.32)	8.71 (2.94)	*p* < 0.001^∗^
Naming task [34]	19.76 (0.66)	18.05 (1.73)	*p* < 0.001^∗^

Means and standard deviations are indicated. Group differences were calculated with independent-sample *t*-*tests*. Note: ^∗^ means significant when corrected for multiple comparisons (*p* < 0.0019).

**Table 3:** Difference in demographic variables and WML volume between high and low WML groups.

	Low WML	High WML	*p* difference
*Healthy ageing*			
*N*	13	12	
Age	64.92	70.58	*p* = 0.052
Years of education	10.92	10.08	*p* = 0.621
WML (%)	0.031	0.461	*p* = 0.012^∗^
WML (%) range	0.002-0.084	0.093-1.891	
*Mild cognitive impairment*			
*N*	11	11	
Age	72.82	74.91	*p* = 0.359
Years of education	7.18	9.82	*p* = 0.090
WML (%)	0.158	1.032	*p* = 0.001^∗^
WML (%) range	0.016-0.315	0.381-2.435	

Means are indicated.

**Table 4:** Results of the two-way mixed *ANOVA*s assessing the interaction between WML group and treatment on cognitive performance.

	Low WML			High WML			*pANOVA* for interaction and main effects			*pBox's M*
	Baseline (mean)	Retest (mean)	*N*	Baseline (mean)	Retest (mean)	*N*	*pANOVA* interaction	*pANOVA* treatment	*pANOVA* WML	
*Healthy ageing*										
Phonemic fluency	34	43.08	13	31.42	38.33	12	0.616	0.001^∗^	0.426	0.049^∗^
Semantic fluency	44.54	49.62	13	41.67	43.75	12	0.511	0.124	0.267	0.006^∗^
Digit cancellation	55.23	54.46	13	52.75	54.58	12	0.104	0.496	0.475	0.756
Similarities	20.38	22.54	13	20.75	21.67	12	0.372	0.034	0.862	0.072
Rey figure—memory	16.77	21.73	13	15.96	16.75	12	0.103	0.028	0.203	0.54
Stroop—time interference	18.04	17.08	13	20.33	21.13	12	0.399	0.934	0.257	0.524
Prose—immediate memory	9.38	12.77	13	8.08	10.75	12	0.629	<0.001^∗^	0.206	0.91
Prose—delayed memory	12.69	16.38	13	10.83	12.83	12	0.193	<0.001^∗^	0.105	0.421
Paired associate learning	13.27	14.46	13	12.54	14.26	12	0.844	0.096	0.7	0.715
*MCI*										
Phonemic fluency	25.45	25.73	11	31.09	30.45	11	0.725	0.666	0.221	0.755
Semantic fluency	29.36	30.09	11	27.73	28	11	0.861	0.701	0.606	0.555
Digit cancellation	44.18	46.55	11	45.82	50.27	11	0.424	0.015	0.457	0.902
Similarities	18.09	16.73	11	20.09	20	11	0.434	0.372	0.194	0.653
Rey figure—memory	7.23	9.68	11	8.41	10.32	11	0.76	0.022	0.661	0.356
Stroop—time interference	48.23	58.09	11	36.15	29.6	10	0.113	0.741	0.107	<0.001^∗^
Prose—immediate memory	5.45	7	11	6.64	8.73	11	0.748	0.042	0.212	0.048^∗^
Prose—delayed memory	5.55	7.18	11	8.27	9.45	11	0.737	0.048	0.085	0.981
Paired associate learning	8.23	8.77	11	9.18	9.46	11	0.729	0.305	0.534	0.688

Note: ^∗^ indicates significance when corrected for multiple comparisons (*p* < 0.0028). Significant *Box's M* (*p* < 0.05) indicates violation of the assumption of homogeneity of covariance matrices. Stroop—time interference: lower value indicates better performance.

**Table 5:** Results of the independent-sample *Mann-WhitneyUtests* assessing the difference in cognitive change scores between high and low WML groups.

	Low WML			High WML			Median change scores		*p* effect of WML on change scores
	Baseline (median)	Retest (median)	*N*	Baseline (median)	Retest (median)	*N*	Low WML	High WML	
*Healthy ageing*									
Token task	35.5	34.75	12	36	35.5	12	0	0	7.99
Raven's progressive matrices	33	34	12	28.5	31.5	12	0.5	2	0.376
Rey figure—copy	34.5	36	12	33	34.5	12	0	1.5	0.32
Stroop—error interference	0	0	12	0	0	12	0	0	0.936
Corsi span	5	5	12	4.5	5	12	0	0	0.65
Digit span—forwards	6	6	12	6	6	12	0	0	0.503
Digit span—backwards	4.5	5.5	12	4	4	12	0	0.5	1
Naming task	20	20	12	20	20	12	0	0	0.538
*MCI*									
Token task	33.5	32.5	11	34	33	10	0	0	0.847
Raven's progressive matrices	24	22	11	27.5	28.5	10	-1	1	0.898
Rey figure—copy	29	28	11	29.25	31.25	10	0	1.75	0.171
Stroop—error interference	3	1.5	11	0.5	0	10	1	0	0.468
Corsi span	4	4	11	4	4	10	0	0	0.133
Digit span—forwards	5	6	11	6	6	10	0	0	0.519
Digit span—backwards	4	3	11	4	4	10	0	0	0.478
Naming task	19	20	11	18	19	10	1	0	0.949

Note: ^∗^ indicates significance when corrected for multiple comparisons (*p* < 0.0028). Stroop—error interference: lower value indicates better performance.

**Table 6:** Effect of WML-by-timepoint interaction on extracted functional networks.

Cluster level *pFWE*	Cluster extent (voxels)	*Z* score at local maximum	Hemisphere	Brodmann area	Talairach coordinates			Brain region
					*x*	*y*	*z*	
*Healthy controls: positive interaction between vascular burden group and timepoint on functional connectivity of the posterior DMN*								
0.006	462	4.65	R	7	28	-57	54	Precuneus
		4.29	R	7	34	-56	54	Superior parietal lobule
		3.92	R	7	18	-48	54	Precuneus
		3.54	R	40	32	-42	52	Inferior parietal lobule
		3.46	R	7	20	-63	57	Superior parietal lobule
		3.37	R	40	36	-44	54	Inferior parietal lobule
		3.18	R	40	36	-50	47	Inferior parietal lobule
0.031	351	4.4	L	7	-26	-56	47	Superior parietal lobule
		3.98	L	40	-40	-36	50	Inferior parietal lobule
		3.9	L	7	-18	-50	43	Precuneus
		3.66	L	40	-28	-36	53	Postcentral gyrus
		3.5	L	40	-36	-40	55	Inferior parietal lobule
		2.93	L	7	-22	-51	58	Superior parietal lobule
		2.64	L	7	-34	-54	52	Superior parietal lobule
*MCI: positive interaction between vascular burden group and timepoint on functional connectivity of the salience network*								
0.036	358	3.54	R	^∗^	34	-13	12	Claustrum
		3.36	R	13	38	-3	13	Insula
		3.33	R	13	38	9	16	Insula
		3.49	R	^∗^	34	10	7	Claustrum
		2.98	R	^∗^	34	4	9	Claustrum
		2.79	R	13	32	-22	20	Insula
*All: interaction between vascular burden and timepoint on functional connectivity of the posterior DMN in participants with low CR*								
0.013	277	3.83	R	24	16	4	37	Cingulate gyrus
		3.77	R	24	4	2	37	Cingulate gyrus
		3.50	R	24	18	-13	43	Cingulate gyrus

Note: only clusters surviving corrected threshold reported. FWE: family-wise error; R: right; L: left.

## Data Availability

The MRI and neuropsychology data used to support the findings of this study have not been made available because the authors have no permission from participants to share their data with researchers not part of the core team of the principal investigator (AV).
